# Electrochemical Performance of Li Metal Anodes in Conjunction with LLZO Solid-State Electrolyte

**DOI:** 10.1021/accountsmr.5c00124

**Published:** 2025-05-20

**Authors:** Kostiantyn V. Kravchyk, Matthias Klimpel, Huanyu Zhang, Maksym V. Kovalenko

**Affiliations:** † Laboratory for Thin Films and Photovoltaics, Empa − Swiss Federal Laboratories for Materials Science and Technology, Überlandstrasse 129, CH-8600 Dübendorf, Switzerland; ‡ Laboratory of Inorganic Chemistry, Department of Chemistry and Applied Biosciences, 27219 ETH Zürich, Vladimir-Prelog-Weg 1, CH-8093 Zürich, Switzerland; § SKKU Institute of Energy Science and Technology (SIEST), Sungkyunkwan University (SKKU), 2066, Seobu-ro, Jangan-gu, Suwon, Gyeonggi-do 16419, Republic of Korea

Since the discovery of Li_7_La_3_Zr_2_O_12_ (LLZO) as a solid-state electrolyte (SSE) capable of replacing flammable organic electrolytes in Li-ion batteries and enabling the use of Li-metal anodes,^1^ research into LLZO-based solid-state batteries (SSBs) has advanced at an impressive pace.^2–5^ Initially, studies focused primarily on understanding Li-ion transport mechanisms to maximize ionic conductivity.^6–8^ Over time, this has evolved into a broad research field encompassing various aspects of LLZO SSEs, including their electrochemical stability window,^9–12^ compatibility with existing cathode chemistries,^13,14^ chemical stability under ambient conditions,^15–17^ and the development of cost-effective synthesis methods.^18,19^ Furthermore, the challenge of Li dendrite formation, observed early on, even in single crystals, has led to various mitigation strategies.^20–23^ These include reducing the electronic conductivity of LLZO grain boundaries,^24,25^ improving surface cleanliness,^26^ developing Li/LLZO interfacial layers,^27^ and employing porous LLZO microstructures.^28^

Despite numerous reviews on Li/LLZO modification strategies to mitigate Li dendrites,^29–31^ it remains unclear what level of electrochemical performance has been achieved after *ca*. 15 years of research. More importantly, there is still no definitive assessment of whether this performance meets commercial standards for conventional Li-ion batteries. This viewpoint aims to bridge this gap by providing a comprehensive assessment of the electrochemical performance of Li metal anodes in combination with LLZO solid-state electrolytes. Our analysis focuses on four key parameters that we consider critical to electrochemical performance: current density, cumulative areal capacity, areal capacity, and LLZO thickness. Furthermore, using reported values of areal capacity and LLZO thickness from Li/LLZO/Li symmetrical cells, we evaluate the state-of-the-art achievable gravimetric and volumetric energy densities of LLZO-based garnet batteries and compare them with those of conventional Li-ion batteries.

Although there are multiple ways to compile all four parameters simultaneously, we chose to present them similarly to the approach used by Albertus et al.^32^ in their Nature Energy paper on Li metal anodes, which we find particularly informative.

The first parameter, shown on the *x*-axis, is the plating current density. This represents the rate at which Li plating and stripping occur, essentially indicating how quickly a battery can be charged or discharged under dendrite-free conditions. The second parameter, shown on the *y*-axis, is the cumulative capacity of plated/stripped Li over cycling. This is the total amount of Li plated across all cycles and is obtained by multiplying the capacity plated per cycle by the number of cycles. Although extracting cumulative capacity values from published data may seem straightforward, several key considerations must be addressed. To determine the actual cumulative capacity for a given LLZO system, whether in symmetrical or full-cell configurations, one must account for the plating/stripping duration until either a short circuit occurs due to dendrite formation or a significant increase in voltage polarization is observed, typically caused by void formation at the Li/LLZO interface. In this analysis, we included only cumulative capacity data that met these conditions across all reviewed studies on LLZO SSEs. This approach allowed us to exclude cases where no sharp voltage drop was reported—an indication, in our view, of soft shorting in symmetrical cells, which can enable seemingly indefinite cycling. Soft shorting is a common phenomenon in Li/SSE/Li symmetrical cells, where both ionic and electronic transport occur within the solid-state electrolyte.^33^

The third parameter, represented by the size of the data points, is areal capacity, which indicates the amount of lithium plated per cycle. As demonstrated by Kravchyk et al.,^34^ achieving high areal capacity is essential for maximizing the energy density of LLZO-based SSBs. It is also important to consider not just the absolute amount of Li plated/stripped per cycle but also the fraction of plated lithium relative to the total lithium available in the assembled cell. In other words, from an energy density perspective, a crucial metric is how much of the available lithium is actively utilized during cycling. However, since most studies do not report details on the lithium thickness used, we were unable to include this parameter in our analysis.

The fourth key parameter is the thickness of the LLZO separator, as a thinner separator directly contributes to higher energy density in SSBs. Additional important factors not shown in Figure 1 include temperature, applied pressure, and the interfacial resistance at the Li/LLZO interface.

**Figure 1:**
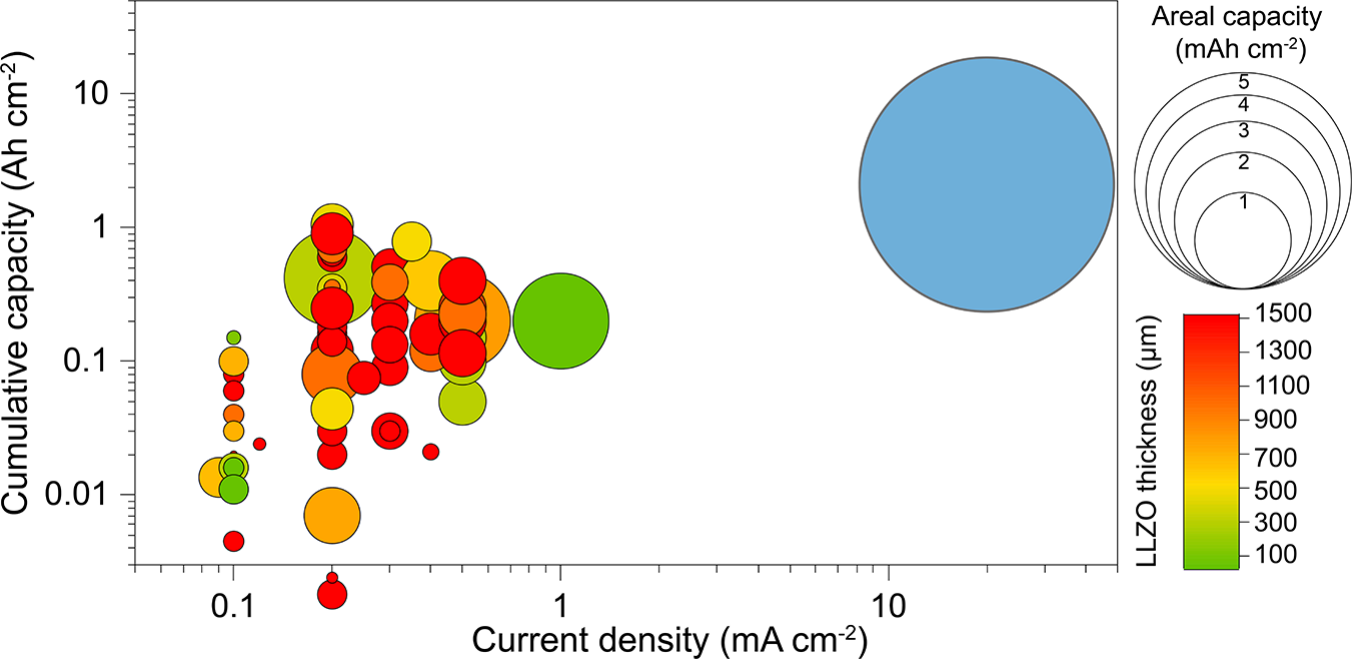
Depiction of published data on the electrochemical cycling of a Li metal anode in conjunction with an LLZO solid electrolyte in a Li/LLZO/Li symmetric cell configuration. Detailed information for each data point shown in Figure 1 can be found in Supporting Information Table S1. The blue circle represents the electrochemical performance of a Panasonic NCR18650GA Li-ion battery with 6.9 mAh cm^–2^ electrodes operated at a discharge current density of 20 mA cm^–2^ (2.9 C) and capable of retaining at least 80% of its initial capacity over 300 cycles (equivalent to a cumulative capacity of 2.1 Ah cm^–2^).

Lastly, it is important to note that the presented data primarily reflect the electrochemical performance of Li/LLZO/Li symmetrical cells rather than full cells (Tables S1–S4). This is because the majority of published studies on Li metal anodes with LLZO solid-state electrolytes focus on symmetrical cell configurations rather than full-cell setups. Moreover, the development of LLZO-based full cells remains at an early stage, which currently limits the use of high cathode loadings and C rates (Table S3), thereby constraining the evaluation of Li metal anode performance with LLZO SSE. However, in principle, any performance achieved in a symmetrical cell should also be attainable in a full cell and can be viewed as a baseline, representing the minimum achievable electrochemical performance for a given system.

The analysis of the data summarized in Figure 1, presented as colored circles of varying diameters representing LLZO separator thickness and areal capacity limitations per half-cycle, allows us to draw several key conclusions. Despite extensive research efforts in recent years to mitigate the formation and penetration of Li dendrites in LLZO SSEs, the overall electrochemical performance of Li metal anodes in combination with LLZO remains far below the commercial requirements for batteries. Ideally, their performance should be at least comparable to that of conventional Li-ion batteries. For instance, one of the best-performing LLZO systems, featuring a Zn interfacial layer, demonstrated a cumulative capacity of 150 mAh cm^–2^ at a current density of 0.5 mA cm^–2^. However, this corresponds to only 300 cycles at an areal capacity limit of 0.25 mAh cm^–2^.^35^ In contrast, commercial Panasonic Li-ion batteries achieve 14 times higher cumulative capacity operated at the higher current density, as indicated by the blue circle in Figure 1. Moreover, this comparison does not even account for the significantly lower areal capacity limits used in LLZO systems – at least 5–6 times lower than the typical areal capacity of commercial electrodes (>3.5 mAh cm^–2^) – as well as the substantial thickness of LLZO separators and the unknown thickness of the Li metal anode. These factors are critical in determining the overall energy density of LLZO-based SSBs. Our previous analysis of theoretical energy densities for LLZO-based batteries demonstrated that achieving energy densities comparable to state-of-the-art Li-ion batteries would require LLZO separators thinner than 50 μm, combined with high areal capacity cathodes (>3 mAh cm^–2^).^34^ However, given the high separator thickness and low areal capacity limits typically used in experiments (Table S1), most tested LLZO systems in symmetrical cell configuration are estimated to achieve energy and power densities in the range of 0.01–130 Wh kg^–1^ and 0.01–130 W kg^–1^ (Table S1, S2; Figures S1, S2), far below the 250 Wh kg^–1^ benchmark for commercial Li-ion batteries. In the case of reported LLZO systems in full cell configuration, their energy densities have been estimated not to exceed 25 Wh kg^–1^, taking into account very low cathode loadings and thick LLZO separators (Tables S3, S4). In this context, one of the most compelling LLZO systems reported by Okur et al.^36^ utilized a 45 μm thin LLZO separator, theoretically capable of achieving an energy density of ∼ 260 Wh kg^–1^ (assuming a 10 μm thin Li metal anode and a LiNi_0.8_Mn_0.1_Co_0.1_O_2_ (NMC811) cathode with 28 vol % Li_6_PS_5_Cl (LPSCl) catholyte, 2 vol % carbon additives, and loading of 25 mg_NMC811_ cm^–2^, cathode areal capacity of 5 mAh cm^–2^). However, the reported Li/LLZO/Li symmetrical cell exhibited limited cycling performance, with a cumulative capacity of 200 mAh cm^–2^, corresponding to only 100 charge/discharge cycles.

It is important to clarify that when discussing the published electrochemical data on Li metal anodes in conjunction with LLZO SSE separators, we refer specifically to the performance of pure Li metal anodes, excluding so-called Li/LLZO anodes based on porous LLZO membranes.^28,37–43^ These porous LLZO-based anodes should be categorized separately because they function as ″host″ structures, where the porous LLZO matrix accommodates metallic Li within its pores. This configuration significantly reduces both volumetric and gravimetric energy density. For example, a Li/LLZO anode utilizing a 50% porous LLZO membrane filled with metallic Li has half the volumetric energy density of pure Li (1031 mAh cm^–3^ vs. 2062 mAh cm^–3^). Notably, its gravimetric capacity (*ca*. 350 mAh g^–1^) is also much lower than that of pure Li (3861 mAh g^–1^) and is comparable to that of graphite (372 mAh g^–1^). However, the employment of these anodes leads to significantly lower overall energy densities of SSBs. For instance, an SSB incorporating a 10 μm-thick LLZO separator, a 5 mAh cm^–2^ NMC811 cathode (with 28 vol % LPSCl catholyte and 2 vol % carbon additives), and a 50 μm-thin Li/porous-LLZO anode capable of accommodating 5 mAh cm^–2^ of metallic Li would achieve an energy density of only 275 Wh kg^–1^. In contrast, an identical battery configuration without a porous LLZO-based anode would attain a much higher energy density of 338 Wh kg^–1^, a 23% increase in energy density. Nevertheless, despite these energy density limitations, batteries based on porous LLZO membranes offer a key advantage: their enlarged Li/LLZO interfacial contact area significantly enhances ultrafast charge/discharge capabilities.^37^ In this regard, porous LLZO-based batteries come closest to meeting commercial requirements for Li metal all-solid-state batteries. However, categorizing them strictly as ″Li metal batteries″ should be done with caution, given the fundamental differences described above with Li metal anode.

## Summary and Outlook

The design of the Li metal anode in garnet-based solid-state batteries (SSBs) remains undeveloped. While various strategies have been proposed to mitigate Li dendrite formation, the issue persists. Reported tests on LLZO-based symmetrical and full cells typically show cumulative capacities far below commercial requirements. Furthermore, the common practice of presenting only the best-performing cells, while partially or completely excluding poorly performing cells, suggests that the actual state-of-the-art performance of Li-metal anodes with LLZO SSEs may be even worse than shown in Figure 1. In this context, we highlight the most critical issues that, in our view, hinder the development of Li metal/LLZO anodes with commercially relevant electrochemical performance.

First, studies often present data from a single cell without disclosing how many additional tests were conducted under identical conditions or their corresponding results.

Second, a significant issue arises when the performance of soft-shortened cells is reported as achievable cycling stability. In many cases, there is no clear evidence that the tested cells are not soft-shortened. This can be verified, for example, by demonstrating a sharp drop in the potential of a symmetrical cell to 0 V or by providing electrochemical impedance spectroscopy data before and after cycling, assuming no hard shorting occurs under the given conditions. Notably, soft-shortened cells may exhibit superior electrochemical performance, but this does not reflect the actual achievable performance of the reported system.

Third, reproducibility remains a major challenge. A careful review of the literature reveals that experimental sections often lack crucial details about cell preparation. Key parameters such as Li thickness, LLZO separator thickness, and the temperature–pressure conditions during cycling are frequently omitted. To accelerate the development of Li garnet-based SSBs, we emphasize the need to report these parameters—not only to ensure reproducibility, but also to enable meaningful comparative analysis.

Furthermore, establishing standardized electrochemical characterization protocols (which still require consensus among researchers) would enhance clarity regarding achieved performance levels and their alignment with commercial requirements for specific applications. More importantly, transparent and well-documented data could significantly accelerate development by enabling machine learning and high-throughput screening approaches, ultimately facilitating the commercialization of Li garnet SSBs.

Finally, a fourth issue, often overlooked but equally important, concerns the mechanical properties of LLZO SSEs. LLZO is highly brittle, has low fracture toughness, and is prone to crack formation, which can lead to Li-metal dendrite growth during battery operation. In this context, most studies use millimeter-thick pellets to evaluate the electrochemical performance of Li metal in combination with LLZO. However, these results may not be directly reproducible with much thinner and significantly more fragile LLZO membranes. To bridge this gap, we encourage researchers working on Li-garnet SSBs to prioritize LLZO membranes with thicknesses of 20–50 μm. Investigating such thin membranes will provide early insights into critical technical challenges, including their manufacturing scalability, Li/LLZO interface engineering, and optimized battery design.

## Supplementary Material


