# Supplementary material for: Electrochemical Performance of Li Metal Anodes in Conjunction with LLZO Solid-State Electrolyte
Source: Acc Mater Res. 2025 May 20;6(7):794–8. doi: 10.1021/accountsmr.5c00124 (PMC12305636; doi:10.1021/accountsmr.5c00124)
Supplement: Supplementary file 1 [file mr5c00124_si_001.pdf]

Supporting Information for

On the electrochemical performance of the Li metal anodes in conjunction with the LLZO solid-state electrolyte

Kostiantyn V. Kravchyk,[1,2] Matthias Klimpel[1,2] Huanyu Zhang,[1,2] and Maksym V. Kovalenko[1,2,3*]*

[1] Laboratory for Thin Films and Photovoltaics, Empa – Swiss Federal Laboratories for Materials Science and Technology, Überlandstrasse 129, CH-8600 Dübendorf, Switzerland

[2] Laboratory of Inorganic Chemistry, Department of Chemistry and Applied Biosciences, ETH Zürich, Vladimir-Prelog-Weg 1, CH-8093 Zürich, Switzerland

[3]SKKU Institute of Energy Science and Technology (SIEST), Sungkyunkwan University (SKKU), 2066, Seobu-ro, Jangan-gu, Suwon, Gyeonggi-do 16419, Republic of Korea

Corresponding Authors:

*E-mails: kostiantyn.kravchyk@empa.ch and mvkovalenko@ethz.ch

Table S1. Summary of published data on the electrochemical cycling of a Li metal anode in conjunction with an LLZO solid electrolyte in a Li/LLZO/Li symmetric cell configuration.

Li/LLZO interface strategy approach	Current density, mA cm^{-2}	Areal capacity limit, mAh cm^{-2}	Number of cycles	Cumulative capacity, Ah cm^{-2}	Temperature, °C	Pressure, MPa	LLZO thickness, µm	Interfacial resistance of Li/LLZO interface, Ohm cm^2	Gravimetric energy density, Wh kg^{-1}	Volumetric energy density, Wh L^{-1}	Gravimetric power density, W kg^{-1}	Volumetric power density, W L^{-1}	Reference
**Interfacial layer (Au)**	0.5	1	100	0.2	50	10	800	-	9.15	44.15	4.61	22.37	[1]
**Interfacial layer (Au)**	0.09	0.18	37	0.0135	25	-	650	380	2.05	9.88	1.03	4.96	[2]
**Interfacial layer (Ag)**	0.2-0.4	0.033 – 0.066	210	0.021	25	-	-	51	0.16	0.81	1.98	9.83	[3]
**Interfacial layer (Ag)**	0.5	0.25	100	0.05	25	-	300	178	6.13	27.64	12.12	54.39	[4]
**Interfacial layer (Ag)**	0.2	0.1	100	0.02	25	-	-	66	0.50	2.47	0.99	4.93	[5]
**Interfacial layer (Ag-WSe_2)**	0.1	0.05	600	0.06	25	-	-	25	0.25	1.23	0.50	2.47	[6]
**Interfacial layer (Al)**	0.2	1	210	0.42	25	-	300	75	23.69	105.36	4.92	22.19	[7]
**Interfacial layer (Al)**	0.2	0.1	3000	0.6	25	-	1500	1	0.50	2.47	0.99	4.93	[8]
**Interfacial layer (Al_2O_3)**	0.2	0.1	90	0.0018	25	-	-	34	0.50	2.47	0.99	4.93	[9]

Li/LLZO interface strategy approach	Current density, mA cm^{-2}	Areal capacity limit, mAh cm^{-2}	Number of cycles	Cumulative capacity, Ah cm^{-2}	Temperature, °C	Press ure, MPa	LLZO thickness, µm	Interfacial resistance of Li/LLZO interface, Ohm cm^2	Gravimetric energy density, Wh kg^{-1}	Volumetric energy density, Wh L^{-1}	Gravimetric power density, W kg^{-1}	Volumetric power density, W L^{-1}	Reference
**Interfacial layer (BN)**	0.5	0.25	200	0.1	-	-	300	18	6.13	27.64	12.12	54.39	[10]
**Interfacial layer (C)**	0.3	0.15	900	0.27	25	-	-	26.2	0.74	3.70	1.49	7.38	[11]
**Interfacial layer (C)**	0.1	0.025	1500	0.15	-	-	130	15	1.43	5.64	5.67	22.32	[12]
**Interfacial layer (C_3N_4)**	0.3	0.15	300	0.09	25	-	-	11	0.74	3.70	1.49	7.38	[13]
**Interfacial layer (Cu)**	0.2	0.033	825	0.054	25	-	-	51	0.16	0.81	0.99	4.93	[14]
**Interfacial layer (CuO)**	0.3	0.15	600	0.2	25	-	-	9.8	0.74	3.70	1.49	7.38	[15]
**Interfacial layer (Cu_3N)**	0.1	0.05	1000	0.1	25	-	-	83.4	0.25	1.23	0.50	2.47	[16]
**Interfacial layer (Cu_6Sn_5)**	0.25	0.125	300	0.075	25	-	-	168	0.62	3.08	1.24	6.15	[17]
**Interfacial layer (CoO)**	0.3	0.15	1696	0.508	25	-	-	12.3	0.74	3.70	1.49	7.38	[18]
**Interfacial layer (Ge)**	0.1	0.0083	42	0.0007	25	-	-	-	0.04	0.21	0.50	2.47	[19]
**Interfacial layer (ITO)**	0.2	0.1	800	0.16	30	-	-	32	0.50	2.47	0.99	4.93	[20]

Li/LLZO interface strategy approach	Current density, mA cm^{-2}	Areal capacity limit, mAh cm^{-2}	Number of cycles	Cumulative capacity, Ah cm^{-2}	Temperature, °C	Press ure, MPa	LLZO thickness, µm	Interfacial resistance of Li/LLZO interface, Ohm cm^2	Gravimetric energy density, Wh kg^{-1}	Volumetric energy density, Wh L^{-1}	Gravimetric power density, W kg^{-1}	Volumetric power density, W L^{-1}	Reference
**Interfacial layer (LiF)**	0.4	0.2	300	0.12	25	0	1000	12.7	1.49	7.29	2.96	14.52	[21]
**Interfacial layer (Li_3N)**	0.1	0.0083	1260	0.020	25	0	-	180 (60°C)	0.04	0.21	0.50	2.47	[22]
**Interfacial layer ($LiNbO_3$)**	0.12	0.02	200	0.024	25	-	-	58	0.10	0.49	0.60	2.96	[23]
**Interfacial layer (MoS_2)**	0.2	0.1	40	0.008	100	-	1000	14	0.74	3.65	1.49	7.29	[24]
**Interfacial layer (Ni-Al)**	0.2	0.1	600	0.12	25	-	-	35	0.50	2.47	0.99	4.93	[25]
**Interfacial layer (PbO)**	0.1	0.05	1000	0.1	25	-	1000	10.5	0.37	1.83	0.74	3.65	[26]
**Interfacial layer (Si)**	0.2	0.016	75	0.0024	25	-	-	-	0.08	0.40	0.99	4.93	[27]
**Interfacial layer (Si_3N_4)**	0.1	0.016	2400	0.08	25	-	-	84.5	0.08	0.40	0.50	2.47	[28]
**Interfacial layer (Si_3N_4)**	0.4	0.4	500	0.4	25	-	600	5	4.92	23.49	4.92	23.49	[29]
**Interfacial layer (SiO_2)**	0.2	0.2	600	0.12	24	-	-	49	0.99	4.93	0.99	4.93	[30]
**Interfacial layer (SiO_2)**	0.3	0.15	100	0.03	30	-	-	3	0.74	3.70	1.49	7.38	[31]

Li/LLZO interface strategy approach	Current density, mA cm^{-2}	Areal capacity limit, mAh cm^{-2}	Number of cycles	Cumulative capacity, Ah cm^{-2}	Temperature, °C	Press ure, MPa	LLZO thickness, µm	Interfacial resistance of Li/LLZO interface, Ohm cm^2	Gravimetric energy density, Wh kg^{-1}	Volumetric energy density, Wh L^{-1}	Gravimetric power density, W kg^{-1}	Volumetric power density, W L^{-1}	Reference
**Interfacial layer (Sn)**	0.5	0.25	500	0.25	25	-	1200	758	1.55	7.64	3.09	15.21	[32]
**Interfacial layer (SnS_2)**	0.1	0.05	1000	0.1	25	-	1000	-	0.37	1.83	0.74	3.65	[33]
**Interfacial layer (SnS_2)**	0.2	0.066	420	0.630	-	-	-	17	0.33	1.63	0.99	4.93	[34]
**Interfacial layer (SnO_2)**	0.2	0.1	900	0.18	25	-	-	153	0.50	2.47	0.99	4.93	[35]
**Interfacial layer (SnO_2)**	0.1	0.05	800	0.08	25	-	-	38	0.25	1.23	0.50	2.47	[36]
**Interfacial layer (Ta_2O_5)**	0.2	0.2	2625	1.05	25	-	450	9	3.29	15.43	3.29	15.43	[37]
**Interfacial layer (Zn-Cu)**	0.2	0.1	300	0.06	25	-	500	-	1.49	7.03	2.96	14.01	[38]
**Interfacial layer (Zn)**	0.35	0.175	2250	0.7875	25	-	500	7.5	2.59	12.27	5.16	24.36	[39]
**Interfacial layer (Zn)**	0.5	0.25	300	0.15	25	-	320	1.9	5.76	26.12	11.38	51.44	[40]
**Interfacial layer (ZnO)**	0.1	0.05	45	0.0045	25	-	-	100	0.25	1.23	0.50	2.47	[41]
**Interfacial layer (molten**	0.4	0.2	400	0.16	30	-	-	13	0.99	4.93	1.98	9.83	[42]

Li/LLZO interface strategy approach	Current density, mA cm^{-2}	Areal capacity limit, mAh cm^{-2}	Number of cycles	Cumulative capacity, Ah cm^{-2}	Temperature, °C	Press ure, MPa	LLZO thickness, µm	Interfacial resistance of Li/LLZO interface, Ohm cm^2	Gravimetric energy density, Wh kg^{-1}	Volumetric energy density, Wh L^{-1}	Gravimetric power density, W kg^{-1}	Volumetric power density, W L^{-1}	Reference
**salt, $NH_4H_2PO_4$)**													
**Interfacial layer (PEO, polyethylene oxide)**	0.2	0.1	700	0.35	90	-	400	-	1.86	8.63	3.70	17.17	[43]
**Interfacial layer (PAA, Polyacrylic acid)**	0.5	0.25	400	0.2	25	-	-	54.5	1.24	6.15	2.47	12.26	[44]
**Interfacial layer (ETPTA-LiPF6)**	0.1	0.05	400	0.04	30	-	1000	88	0.37	1.83	0.74	3.65	[45]
**Interfacial layer (PDMS)**	0.2	0.033	5400	0.3564	25	-	1000	107	0.25	1.21	1.49	7.29	[46]
**Interfacial layer (sulfonated COF)**	0.1 – 3.0	0.05 – 1.5	380	*ca.* 0.1	25	-	-	99	0.25	1.23	0.50	2.47	[47]
**Interfacial layer (FEC-based plastic crystal)**	0.2	0.1	150	0.03	-	-	-	560	0.50	2.47	0.99	4.93	[48]

Li/LLZO interface strategy approach	Current density, mA cm^{-2}	Areal capacity limit, mAh cm^{-2}	Number of cycles	Cumulative capacity, Ah cm^{-2}	Temperature, °C	Pressure, MPa	LLZO thickness, µm	Interfacial resistance of Li/LLZO interface, Ohm cm^{2}	Gravimetric energy density, Wh kg^{-1}	Volumetric energy density, Wh L^{-1}	Gravimetric power density, W kg^{-1}	Volumetric power density, W L^{-1}	Reference
***LLZO surface cleaning (mechanical cleaning: rubbing the SSE pellet on molten Li)**	13.3	0.4	500	0.4	25	-	1000	-	1.06	5.13	1.06	5.13	[49]
**LLZO surface cleaning (thermal treatment)**	0.1	0.1	530	0.1	25	-	700	55	3.45	16.71	1.98	9.59	[50]
**LLZO surface cleaning (thermal treatment)**	0.2	0.35	10	0.007	25	0.4	750	-	3.45	16.71	1.98	9.59	[51]
**LLZO surface cleaning (thermal treatment)**	0.2	0.35	10	0.007	25	3.2	750	-	0.25	1.23	1.49	7.38	[51]
**LLZO surface cleaning**	0.3	0.05	300	0.03	25	-	-	-	2.96	14.52	1.49	7.29	[52]

Li/LLZO interface strategy approach	Current density, mA cm^{-2}	Areal capacity limit, mAh cm^{-2}	Number of cycles	Cumulative capacity, Ah cm^{-2}	Temperature, °C	Pressure, MPa	LLZO thickness, μm	Interfacial resistance of Li/LLZO interface, Ohm cm^2	Gravimetric energy density, Wh kg^{-1}	Volumetric energy density, Wh L^{-1}	Gravimetric power density, W kg^{-1}	Volumetric power density, W L^{-1}	Reference
**(thermal treatment)**													
**LLZO surface cleaning (thermal treatment)**	0.2	0.4	100	0.08	25	0.35	1000	-	4.53	22.43	0.23	1.14	[53]
**LLZO surface cleaning (thermal treatment)**	0.046	0.92	2	0.00018	25	-	-	109	2.47	11.17	2.47	11.17	[54]
**LLZO surface cleaning (thermal treatment with carbon)**	0.1	0.1	80	0.016	25	-	300	-	0.50	2.47	0.99	4.93	[55]
**LLZO surface cleaning (acid treatment)**	0.2	0.1	700	0.14	30	-	-	-	2.96	14.01	2.96	14.01	[56]
**LLZO surface**	0.2	0.2	110	0.044	25	-	500	-	0.99	4.93	0.99	4.93	[57]

Li/LLZO interface strategy approach	Current density, mA cm^{-2}	Areal capacity limit, mAh cm^{-2}	Number of cycles	Cumulative capacity, Ah cm^{-2}	Temperature, °C	Pressure, MPa	LLZO thickness, µm	Interfacial resistance of Li/LLZO interface, Ohm cm^2	Gravimetric energy density, Wh kg^{-1}	Volumetric energy density, Wh L^{-1}	Gravimetric power density, W kg^{-1}	Volumetric power density, W L^{-1}	Reference
**cleaning (acid treatment HCl)**													
**LLZO surface cleaning (acid treatment HCl)**	0.2	0.2	625	0.25	25	-	-	36	1.86	9.10	3.70	18.11	[58]
**LLZO surface cleaning (acid treatment H_3PO_4)**	0.5	0.25	450	0.225	25	-	1000	7.0	1.24	6.15	2.47	12.26	[59]
**LLZO surface cleaning (acid treatment H_3BO_3 + HF)**	0.5	0.25	1200	0.4	25	-	-	9	0.74	3.65	1.49	7.29	[60]
**LLZO surface cleaning ("acid"**	0.2	0.1	3500	0.7	25	-	1000	4.5	1.24	6.15	2.47	12.26	[61]

Li/LLZO interface strategy approach	Current density, mA cm^{-2}	Areal capacity limit, mAh cm^{-2}	Number of cycles	Cumulative capacity, Ah cm^{-2}	Temperature, °C	Pressure, MPa	LLZO thickness, µm	Interfacial resistance of Li/LLZO interface, Ohm cm^2	Gravimetric energy density, Wh kg^{-1}	Volumetric energy density, Wh L^{-1}	Gravimetric power density, W kg^{-1}	Volumetric power density, W L^{-1}	Reference
**treatment $AgNO_3$)**													
**LLZO surface cleaning ("acid" treatment NH_4F)**	0.5	0.25	230	0.115	25	-	-	38.7	0.62	3.08	1.24	6.15	[62]
**LLZO surface cleaning (citric acid treatment)**	0.25	0.125	300	0.075	25	-	-	11.5	0.25	1.23	0.50	2.47	[63]
**LLZO surface cleaning (dopamine in acid treatment)**	0.1	0.05	600	0.06	25	-	-	-	0.01	0.05	0.01	0.03	[64]
**LLZO surface cleaning (liquid electrolyte soaking)**	0.0014	0.002	23	0.000092	30	-	-	-	0.99	4.93	0.99	4.93	[65]

Li/LLZO interface strategy approach	Current density, mA cm^{-2}	Areal capacity limit, mAh cm^{-2}	Number of cycles	Cumulative capacity, Ah cm^{-2}	Temperature, °C	Pressure, MPa	LLZO thickness, µm	Interfacial resistance of Li/LLZO interface, Ohm cm^2	Gravimetric energy density, Wh kg^{-1}	Volumetric energy density, Wh L^{-1}	Gravimetric power density, W kg^{-1}	Volumetric power density, W L^{-1}	Reference
**LLZO surface cleaning (polishing)**	0.2	0.2	2250	0.9	25	-	-	1.7	0.74	3.70	1.49	7.38	[66]
**LLZO surface cleaning (polishing)**	0.3	0.15	400	0.133	25	-	-	28.15	1.12	5.47	2.23	10.91	[67]
**LLZO surface cleaning (polishing)**	0.3	0.15	1300	0.39	25	-	1000	20.9	0.53	2.57	1.06	5.13	[68]
**LLZO surface cleaning (Nanosecond Laser Cleaning)**	0.1	0.05	300	0.03	80	-	700	3.1	125.11	359.63	125.11	359.63	[69]
***LLZO surface cleaning (thermal treatment)**	1	1	200	0.2	75	-	45	45.3	8.92	23.71	17.54	46.76	[70]
**Interfacial layer (PEO)**	0.1	0.05	~50	0.016	65	-	41	594	16.38	45.10	16.38	45.10	[71]

Li/LLZO interface strategy approach	Current density, mA cm^{-2}	Areal capacity limit, mAh cm^{-2}	Number of cycles	Cumulative capacity, Ah cm^{-2}	Temperature, °C	Press ure, MPa	LLZO thickness, µm	Interfacial resistance of Li/LLZO interface, Ohm cm^2	Gravimetric energy density, Wh kg^{-1}	Volumetric energy density, Wh L^{-1}	Gravimetric power density, W kg^{-1}	Volumetric power density, W L^{-1}	Reference
**Interfacial layer (Au)**	0.1	0.05-0.1	~150	0.011	RT	-	44	~30	9.15	44.15	4.61	22.37	[72]
***Interfacial layer (Al_2O_3)**	3	2	>400	>1.2	RT	-	150	30	-	-	-	-	[73]
**Porous microstructure**	0.5	1	75	0.15	-	-	124	-	-	-	-	-	[74]
***Porous microstructure + CNT coating**	3	3	70	0.42	-	-	150	-	-	-	-	-	[75]
***Porous microstructure**	10	1.25	7	0.0175	-	-	154	-	-	-	-	-	[73]
***Porous microstructure**	0.5	0.25	170	0.085	-	-	30	116	-	-	-	-	[76]
***Porous microstructure**	0.4	0.2	180	0.072	75	0	60	133	-	-	-	-	[77]
***Porous microstructure**	0.1	0.1	630	0.126	25	0	60	24.6	-	-	-	-	[78]

Li/LLZO interface strategy approach	Current density, mA cm^{-2}	Areal capacity limit, mAh cm^{-2}	Number of cycles	Cumulative capacity, Ah cm^{-2}	Temperature, °C	Pressure, MPa	LLZO thickness, µm	Interfacial resistance of Li/LLZO interface, Ohm cm^2	Gravimetric energy density, Wh kg^{-1}	Volumetric energy density, Wh L^{-1}	Gravimetric power density, W kg^{-1}	Volumetric power density, W L^{-1}	Reference
***Porous microstructure + carbon coating**	3	6	150	1.8	-	-	90	-	-	-	-	-	[79]
***Porous microstructure by MIEC**	30-100	15-50	50-100	10-100	RT	-	115	~11	-	-	-	-	[80]
**Porous microstructure + Al_2O_3 5 nm + polymer coating on each side**	0.3	0.15	160	0.15	RT	-	105	-	-	-	-	-	[81]
***Porous microstructure + ZnO 10 nm**	0.5	0.5	150	0.075	RT	-	70	60-40	-	-	-	-	[82]
**Panasonic NCR18650GA commercial Li-ion cell**	20	6.9	300	2.07	RT	-	-	-	-	-	-	-	[83]

* No evidence has been provided to confirm the absence of soft shorting in tested cells, such as sharp voltage drops or EIS data before and after cycling.

Calculations of energy and power densities of full cells based on reported data from symmetrical cell measurements (Table S1)

The achievable gravimetric, volumetric energy and power densities were calculated using three parameters from reported Li/LLZO/Li symmetric cell measurements: areal capacity limit (mAh cm^{-2}), current density (mA cm^{-2}) and LLZO separator thickness (µm). As the majority of published papers did not report the thickness of the Li metal anode used, we used a constant value of 10 µm for the Li thickness for all systems. As for the cathode part of the complete cell, it was assumed that the Li/LLZO part of the reported symmetrical cell could hypothetically be combined with the solid state cathode of the same areal capacity. Among the different options for the selection of the specific cathode active material and the catholyte for performing the calculation, we have opted for $LiNi_{0.8}Mn_{0.1}Co_{0.1}O_2$ (NMC811) and Li_6PS_5Cl (LPSCl), considering the high charge storage capacity of 200 mAh g^{-1} and voltage of 3.8 V of the NMC811 active material and the high conductivity, low density (2.0 g cm^{-3}) and ductility of the LPSCl solid electrolyte.
The cathode was assumed to consist of 29 wt% LPSCl catholyte 3.5 wt% carbon black and 67.5 wt% NMC811. It was also assumed that there was no unoccupied pore volume. The thickness of the NMC811 cathode was calculated based on its areal capacity, which was assumed to be the same as the areal capacity limit used in the Li/LLZO/Li symmetric cell measurements.
The calculations were performed considering 100% utilization of the NMC811 active material, an average cell voltage of 3.8 V and the total weight or volume of all cell components. The cell volume was calculated in the fully discharged state, which is the state in which the battery would be assembled. The cell was assumed to consist of 1 layer of Al foil (16 µm) as anode current collector and 1 layer of Cu foil (12 µm) as cathode current collector. The dimensions of the battery were 5.5 cm × 8.5 cm. The summary of all parameters used for the energy and power density calculations can be found in Table S2.

Table S2. Parameters used in calculating energy density of NMC811-LPSCl | LLZO | Li full cells.

**Parameters**	**Unit**	**Value**
Thickness of Al current collector	µm	16
Density of NMC811	g cm^{-3}	4.77
Density of Carbon additives	g cm^{-3}	1.8
Density of LPSCl	g cm^{-3}	2.0
Ratio of NMC-Carbon-LPSCl	-	67.5 : 3.5 : 29 in weight
Theoretical capacity of NMC811	mAh g^{-1}	200
Average voltage	V	3.8
Density of LLZO	g cm^{-3}	5.1
Thickness of Li anode	µm	10
Thickness of Cu current collector	µm	12

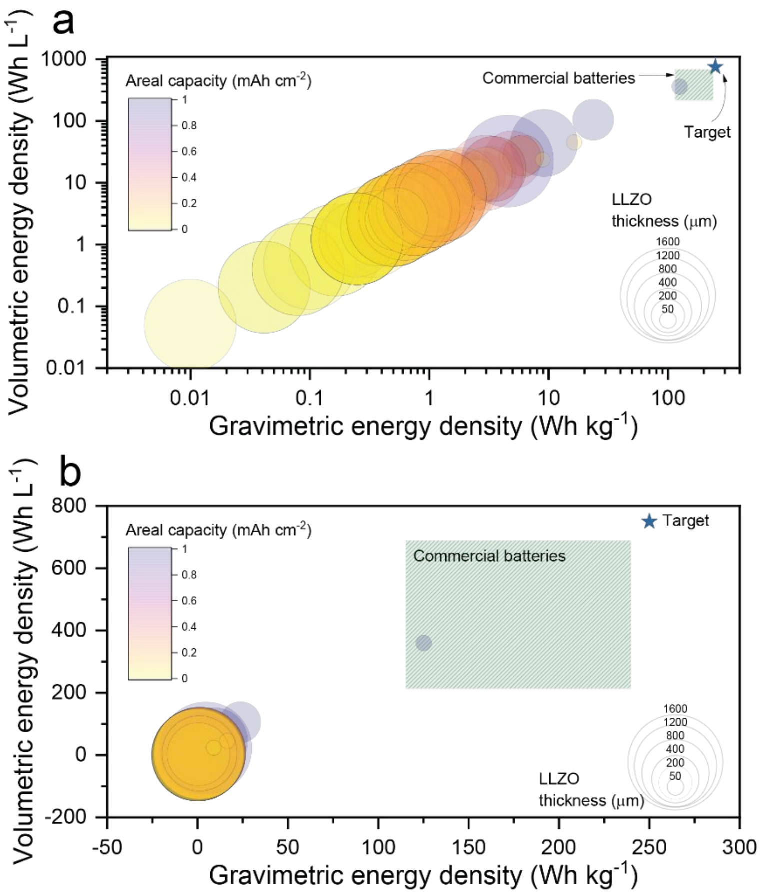

Figure S1. Energy density calculated based on a NMC811-LPSCl/LLZO/Li full cell configuration and published cycling data with LLZO solid electrolytes in a Li/LLZO/Li symmetric cell configuration, including LLZO thickness and capacity limitations. Detailed information for each data point shown in Figure 1 can be found in Supplementary Table 1. The green region represents the electrochemical performance of commercial batteries in Ref.[84] And the target point is 250 Wh kg^{-1} and 750 Wh L^{-1}.

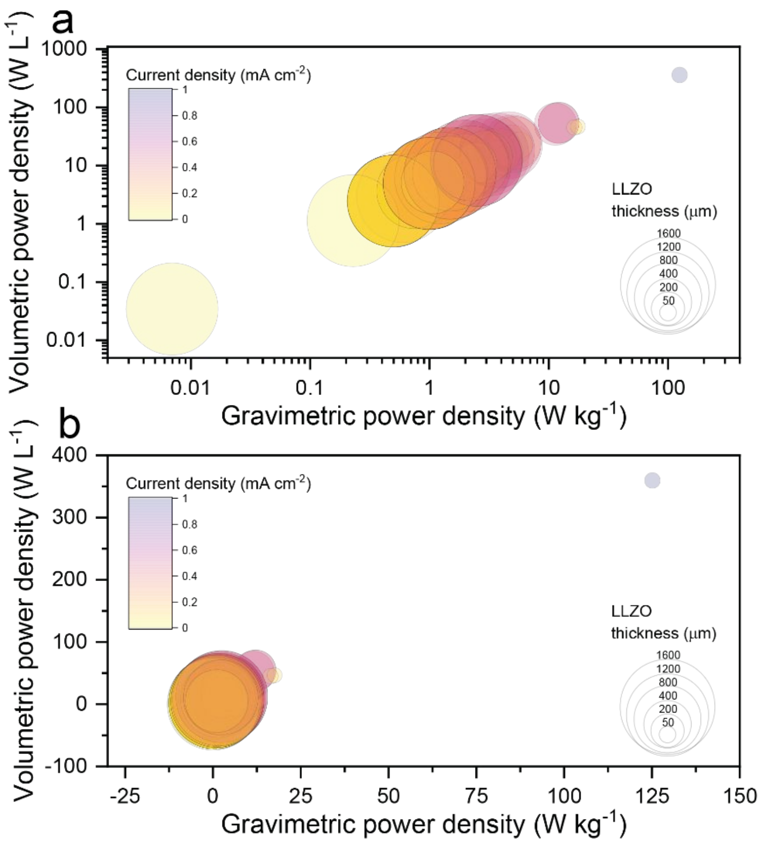

Figure S2. Power density calculated based on a NMC811-LPSCl/LLZO/Li full cell configuration and published cycling data with LLZO solid electrolytes in a Li/LLZO/Li symmetric cell configuration, including LLZO thickness and current density.

Table S3. Summary of published data on the electrochemical cycling of a Li metal anode and different cathode active materials in conjunction with an LLZO solid electrolyte in a Li/LLZO/CAM full cell configuration.

Cathode active material	Catholyte material	Voltage window, V	First cycle discharge capacity, mAh g^{-1}	Loading, mg cm^{-2}	Current density, mA cm^{-2}	Number of cycles	Capacity retention, %	LLZO thickness, µm	Temperature, °C	Pressure, MPa	Reference
**LFP**	$LiPF_6$ in EC/DMC	2.6 – 3.9	149.8	2.0	0.017	100	95	300	25	-	[4]
**LFP**	LiTFSI in DME/DOL	2.7 – 4.0	153	2.4	0.1	500	90	1500	25	-	[6]
**LFP**	$LiPF_6$ in EC/DEC	2.0 – 4.5	132	1.0	0.1	100	91	300	20	-	[7]
**LFP**	LiTFSI in EC/DMC	2.5 – 4.0	133	5.0	0.425	100	> 100	1500	25	-	[8]
**LFP**	LLZO	2.8 – 4.0	128	8.8	0.3	100	93	300	60	-	[10]
**LFP**	$LiPF_6$ in EC/DME/EMC	2.5 – 4.2	135	1.8	0.03	330	87	-	-	-	[11]
**LFP**	$LiPF_6$ in EC/DMC	2.5 – 4.0	150	2.0	0.03	185	92.5	-	25	-	[18]
**LFP**	$LiPF_6$ in EC/DEC	2.4 – 4.0	138	-	1 C	100	100	-	-	-	[19]
**LFP**	$LiPF_6$ in EC/DEC	2.5 – 4.0	150	2.0	0.068	100	99	400	-	-	[20]
**LFP**	LiTFSI in PEO	2.4 – 4.0	136	2.0	0.05	100	90	-	-	-	[22]
**LFP**	-	2.5 – 4.0	157	-	1 C	100	80	-	25	-	[25]
**LFP**	LiTFSI in PEO	2.5 – 4.0	118	5.0	0.3	140	> 100	1000	60	-	[26]
**LFP**	$LiPF_6$ in EC/DEC	2.5 – 4.0	147	2.0	0.068	100	86	-	25	-	[28]

Cathode active material	Catholyte material	Voltage window, V	First cycle discharge capacity, mAh g^{-1}	Loading, mg cm^{-2}	Current density, mA cm^{-2}	Number of cycles	Capacity retention, %	LLZO thickness, µm	Temperature, °C	Pressure, MPa	Reference
**LFP**	LiTFSI in PEO	2.3 – 4.2	146	2.0	0.34	100	97	-	60	-	[29]
**LFP**	$LiPF_6$ in DME/DOL	2.5 – 3.8	148	2.0	0.034	50	> 100	-	-	-	[30]
**LFP**	$LiPF_6$ in EC/DMC/EMC	2.5 – 4.0	127	1.8	0.05	80	95	-	-	-	[31]
**LFP**	-	2.7 – 4.0	152	-	0.1 C	100	> 100	1200	-	-	[32]
**LFP**	$LiPF_6$ in EC/DEC	2.0 – 4.2	156	4.0	0.068	100	90	1000	25	-	[33]
**LFP**	$LiPF_6$ in EC/DEC	2.5 – 3.8	137	1.0	0.05	100	~ 100	-	25	-	[35]
**LFP**	LiTFSI in Succinonitrile	2.4 – 4.0	158	4.0	0.14	100	100	450	25	<2.5	[37]
**LFP**	$LiPF_6$ in EC/DEC	2.5 – 4.1	136	2.0	0.34	50	89	500	25	-	[38]
**LFP**	LiTFSI in PEO	2.5 – 4.0	137	2.0	0.07	200	92	400	90	-	[43]
**LFP**	LiTFSI in Py14TFSI	2.6 – 3.8	130	2.0 – 3.0	~ 0.2	260	89	-	25	-	[47]
**LFP**	LiTFSI in PVDF	2.8 – 4.2	148	2.0	0.034	40	78	-	60	-	[52]
**LFP**	LiTFSI in CPEO	2.5 – 3.8	143	2.0 – 3.0	0.1	50	> 100	300	65	-	[55]
**LFP**	LiTFSI in Py14TFSI	2.8 – 3.8	142	2.0	0.034	150	82	-	30	-	[56]

Cathode active material	Catholyte material	Voltage window, V	First cycle discharge capacity, mAh g^{-1}	Loading, mg cm^{-2}	Current density, mA cm^{-2}	Number of cycles	Capacity retention, %	LLZO thickness, µm	Temperature, °C	Pressure, MPa	Reference
**LFP**	$LiPF_6$ in EC/DMC	2.5 – 4.0	150	4.3	0.095	100	~100	1000	25	-	[59]
**LFP**	LiTFSI in EC/DMC	2.5 – 4.0	132	5.8	1.0	250	90	1000	60	-	[60]
**LFP**	LiTFSI in EC/DMC	-	127	4.3 – 4.6	0.15	320	95	1000	60	-	[61]
**LFP**	$LiPF_6$ in EC/DMC/DEC	2.5 – 4.0	150	5.0	0.17	50	96	1200	25	-	[62]
**LFP**	-	2.5 – 3.8	130	2.0	0.034	100	~ 100	-	25	-	[64]
**LFP**	Ionic Liquid	2.5 – 4.0	137	-	0.5	50	~ 100	800	25	-	[66]
**LFP**	$LiPF_6$ in EC/DMC	2.5 – 4.3	142	-	0.4 C	200	92	1500	25	-	[68]
**LFP**	LiTFSI in PEO	2.8 – 3.8	149	1.5	0.025	100	85	700	40	-	[69]
**LFP**	LiTFSI in Py14TFSI	2.0 – 4.2	146	3.0	0.05	30	65	50*	25	-	[76]
**LFMP**	$LiPF_6$ in ETPTA	2.5 – 4.5	125	2.4	0.04	200	85	1000	30	-	[45]
**LCO**	Liquid electrolyte	2.8 – 4.2	142	1.5	0.042	100	90	150	25	-	[12]
**LCO**	LiTFSI in Py14TFSI	3.2 – 4.3	125	2.0	0.11	300	80	-	-	-	[16]
**LCO**	-	3.0 – 4.3	132	2.0	0.055	100	75	-	-	-	[17]

Cathode active material	Catholyte material	Voltage window, V	First cycle discharge capacity, mAh g^{-1}	Loading, mg cm^{-2}	Current density, mA cm^{-2}	Number of cycles	Capacity retention, %	LLZO thickness, µm	Temperature, °C	Pressure, MPa	Reference
**LCO**	LiTFSI in Py14TFSI	3.0 – 4.2	112	2.3	0.632	2500	77	1000	60	-	[21]
**LCO**	LiTFSI in PP13TFSI	3.0 – 4.2	130	3.2	0.045	150	81	1000	30	-	[42]
**LCO**	$LiPF_6$ in EC/DEC	3.0 – 4.2	115	-	1 C	100	89	500	25	-	[46]
**LMO**	LiTFSI in Py14TFSI	3.0 – 4.5	108	1.8	0.102	100	83	300	25	-	[5]
**NMC111**	LiTFSI in EC/DMC	2.0 – 4.8	162	2.0	0.05	80	87	500	25	-	[23]
**NMC111**	$LiPF_6$ in EC/DEC	3.0 – 4.2	145	-	1 C	100	82	500	25	-	[46]
**NMC532**	LiTFSI in EC/DMC	2.8 – 4.2	152	6.2	0.25	220	70	1000	25	-	[26]
**NMC532**	LiTFSI in Succinonitrile	2.7 – 4.3	143	4.9	0.1	170	~ 100	320	-	-	[40]
**NMC532**	$LiPF_6$ in EC/DMC	-	200	5.0	0.15	120	55	1000	25	-	[59]
**NMC532**	LiTFSI in EC/DMC	2.5 – 4.0	145	3.0	0.2	130	~ 100	1000	25	-	[60]
**NMC532**	LiTFSI in EC/DMC	-	127	7.0	0.28	150	95	1000	25	-	[61]
**NMC532**	$LiPF_6$ in EC/DEC	2.5 – 4.5	153	14	0.224	30	> 100	70*	-	-	[82]

**Cathode active material**	**Catholyte material**	**Voltage window, V**	**First cycle discharge capacity, mAh g^{-1}**	**Loading, mg cm^{-2}**	**Current density, mA cm^{-2}**	**Number of cycles**	**Capacity retention, %**	**LLZO thickness, µm**	**Temperature, °C**	**Pressure, MPa**	**Reference**
**NMC622**	LiTFSI, LiBOB in Succinonitrile	2.5 – 4.4	135	4.0 – 5.0	0.07	4	91	300*	25	-	[72]
**NMC622**	$LiPF_6$ in EC/DEC	2.0 – 4.5	130	10.5	2.3	520	72	115*	25	-	[80]
**NMC811**	LiTFSI in Succinonitrile	3.0 – 4.3	193	4.0	0.16	150	82	450	25	<2.5	[37]
**NMC811**	$LiPF_6$ in EC/DMC	3.0 – 4.3	171	-	0.2 C	100	70	1500	25	-	[68]
**Pyr IHF**	LiFSI in Py14FSI	2.0 – 4.2	173	0.41	0.1	300	80	50	25	-	[70]
**Sulfur**	LiTFSI in DME/DOL	1.0 – 3.5	1532	1.0	-	30	65	300	20	-	[7]
**Sulfur**	LiTFSI, P_2S_5, Li_2S in TEGDME	-	775	1.0	0.836	1000	65	-	25	-	[15]
**Sulfur**	S, Li_2S, LiOTf in DME/DOL	1.8 – 2.85	850	1.0	0.2	60	80	300	65	-	[55]
**Sulfur**	LiTFSI in DME/DOL	1.3 – 3.0	1131	1.0	0.2	200	53	1000	25	-	[59]
**Sulfur**	LiTFSI in Py14TFSI	1.0 – 3.0	1244	5.3	0.22	8	96	200*	-	-	[73]
**Sulfur**	LiTFSI in DME/DOL	1.0 – 3.5	645	7.5	0.2	32	73	200*	-	-	[81]

**Cathode active material**	**Catholyte material**	**Voltage window, V**	**First cycle discharge capacity, mAh g^{-1}**	**Loading, mg cm^{-2}**	**Current density, mA cm^{-2}**	**Number of cycles**	**Capacity retention, %**	**LLZO thickness, µm**	**Temperature, °C**	**Pressure, MPa**	**Reference**
**SnS_2**	SnS_2	0.01 – 3.0	781	0.8	0.18	100	85	800	-	-	[34]

* LLZO electrolytes with porous structures.

Calculations of full cell energy densities based on reported data from full cell measurements (Table S3)

The achievable energy densities were calculated taking into account all reported parameters such as the thickness of the LLZO separator, the composition of the cathode and the areal capacity of the cathode. As the majority of published papers did not report the thickness of the Li metal anode used and cathode composition, we used a constant value of Li thickness of 10 μm for all systems. The unknown cathode compositions were fixed to, cathode activate materials (CAM) : carbon black : catholyte = 67.5 : 3.5 : 29 in weight. The calculations were performed considering 100% active material utilisation, average cell voltage at given C-rate and total weight or volume of all cell components. The cell volume was calculated in the fully discharged state for Li-containing cathodes or in the charged state for Li-free cathodes (sulphur), which are the states in which the batteries would be assembled. The cell was assumed to consist of 1 layer of Al foil (16 μm) as anode current collector and 1 layer of Cu foil (12 μm) as cathode current collector. A summary of all the parameters used in the energy density calculations is given in Table S4.

Table S4. Parameters used in calculating energy density of published full cells.

**Parameters**	**Unit**	**Value**
Thickness of Al current collector	μm	16
Density of Carbon additives	g cm^{-3}	1.8
Density of liquid catholytes	g cm^{-3}	1.5
Ratio of CAM:carbon black:catholyte	-	67.5 : 3.5 : 29 in weight
Density of LLZO	g cm^{-3}	5.1
Thickness of Li anode	μm	10
Thickness of Cu current collector	μm	12

Panasonic NCR18650GA commercial battery cell data

Estimating the performance of commercial battery cells poses challenges due to limited disclosure of electrochemical cycling data by manufacturers. In our study, a comparison of 18650 cylindrical cells using a consistent electrode area of 500 cm^2 was conducted, as illustrated in Figure 5 of Ref.[84] Specific metrics such as the cell's specific capacity (3450 mAh) and discharge current (10 A) were sourced from the Panasonic NCR18650GA datasheet and subsequently converted to areal capacity and current density.[83] Cumulative capacity was evaluated over 300 cycles to assess cycling performance.

REFERENCES

[1] S. Matsuda, K. Nakamura, *ACS Appl. Energy Mater.* **2020**, *3*, 11113-11118.

[2] C.-L. Tsai, V. Roddatis, C. V. Chandran, Q. Ma, S. Uhlenbruck, M. Bram, P. Heitjans, O. Guillon, *ACS Appl. Mater. Interfaces* **2016**, *8*, 10617-10626.

[3] X. Xiang, Y. Zhang, H. Wang, C. Wei, F. Chen, Q. Shen, *J. Electrochem. Soc.* **2021**, *168*, 060515.

[4] Y. Luo, W. Feng, Z. Meng, Y. Wang, X. Jiang, Z. Xue, *Electrochim. Acta* **2021**, *397*, 139285.

[5] W. Feng, X. Dong, P. Li, Y. Wang, Y. Xia, *J. Power Sources* **2019**, *419*, 91-98.

[6] Z. Sun, Y. Lai, N. Lv, L. Jiang, M. Jia, J. Li, W. Bao, F. Liu, *J. Power Sources* **2020**, *468*, 228379.

[7] K. Fu, Y. Gong, B. Liu, Y. Zhu, S. Xu, Y. Yao, W. Luo, C. Wang, S. D. Lacey, J. Dai, Y. Chen, Y. Mo, E. Wachsman, L. Hu, *Science Advances* **2017**, *3*, e1601659.

[8] Y. Lu, X. Huang, Y. Ruan, Q. Wang, R. Kun, J. Yang, Z. Wen, *J. Mater. Chem. A* **2018**, *6*, 18853-18858.

[9] X. Han, Y. Gong, K. Fu, X. He, G. T. Hitz, J. Dai, A. Pearse, B. Liu, H. Wang, G. Rubloff, Y. Mo, V. Thangadurai, E. D. Wachsman, L. Hu, *Nat. Mater.* **2017**, *16*, 572-579.

[10] S. Rajendran, A. Pilli, O. Omolere, J. Kelber, L. M. R. Arava, *Chem. Mater.* **2021**, *33*, 3401-3412.

[11] C. Cui, Q. Ye, C. Zeng, S. Wang, X. Xu, T. Zhai, H. Li, *Energy Storage Mater.* **2022**, *45*, 814-820.

[12] J. Zhang, J. Li, H. Zhai, G. Tan, X. Tang, *ACS Appl. Energy Mater.* **2020**, *3*, 6139-6145.

[13] Y. Huang, B. Chen, J. Duan, F. Yang, T. Wang, Z. Wang, W. Yang, C. Hu, W. Luo, Y. Huang, *Angew. Chem. Int. Ed.* **2020**, *59*, 3699-3704.

[14] X. Xiang, S. Cao, F. Chen, Q. Shen, L. Zhang, *J. Electrochem. Soc.* **2019**, *166*, A3028.

[15] J. Duan, L. Huang, T. Wang, Y. Huang, H. Fu, W. Wu, W. Luo, Y. Huang, *Adv. Funct. Mater.* **2020**, *30*, 1908701.

[16] H. Huo, Y. Chen, R. Li, N. Zhao, J. Luo, J. G. Pereira da Silva, R. Mücke, P. Kaghazchi, X. Guo, X. Sun, *Energy & Environmental Science* **2020**, *13*, 127-134.

[17] W. Feng, X. Dong, Z. Lai, X. Zhang, Y. Wang, C. Wang, J. Luo, Y. Xia, *ACS Energy Lett.* **2019**, *4*, 1725-1731.

[18] G. Lu, Z. Dong, W. Liu, X. Jiang, Z. Yang, Q. Liu, X. Yang, D. Wu, Z. Li, Q. Zhao, X. Hu, C. Xu, F. Pan, *Science Bulletin* **2021**, *66*, 1746-1753.

[19] W. Luo, Y. Gong, Y. Zhu, Y. Li, Y. Yao, Y. Zhang, K. Fu, G. Pastel, C.-F. Lin, Y. Mo, E. D. Wachsman, L. Hu, *Adv. Mater.* **2017**, *29*, 1606042.

[20] J. Lou, G. Wang, Y. Xia, C. Liang, H. Huang, Y. Gan, X. Tao, J. Zhang, W. Zhang, *J. Power Sources* **2020**, *448*, 227440.

[21] S. Tang, G. Chen, F. Ren, H. Wang, W. Yang, C. Zheng, Z. Gong, Y. Yang, *J. Mater. Chem. A* **2021**, *9*, 3576-3583.

[22] H. Xu, Y. Li, A. Zhou, N. Wu, S. Xin, Z. Li, J. B. Goodenough, *Nano Lett.* **2018**, *18*, 7414-7418.

[23] G. V. Alexander, M. S. Indu, S. Kamakshy, R. Murugan, *Electrochim. Acta* **2020**, *332*, 135511.

[24] J. Fu, P. Yu, N. Zhang, G. Ren, S. Zheng, W. Huang, X. Long, H. Li, X. Liu, *Energy & Environmental Science* **2019**, *12*, 1404-1412.

[25] W. Liu, G. Lu, Z. Yang, Q. Zhao, X. Hu, D. Wu, Z. Li, R. Wang, S. Sun, C. Xu, *Chem. Commun.* **2021**, *57*, 10214-10217.

[26] M. Cai, Y. Lu, L. Yao, J. Jin, Z. Wen, *Chem. Eng. J.* **2021**, *417*, 129158.

[27] W. Luo, Y. Gong, Y. Zhu, K. K. Fu, J. Dai, S. D. Lacey, C. Wang, B. Liu, X. Han, Y. Mo, E. D. Wachsman, L. Hu, *J. Am. Chem. Soc.* **2016**, *138*, 12258-12262.

[28] A. Baniya, A. Gurung, J. Pokharel, K. Chen, R. Pathak, B. S. Lamsal, N. Ghimire, R. S. Bobba, S. I. Rahman, S. Mabrouk, A. L. Smirnova, K. Xu, Q. Qiao, *ACS Appl. Energy Mater.* **2022**, *5*, 648-657.

[29] M. Du, Y. Sun, B. Liu, B. Chen, K. Liao, R. Ran, R. Cai, W. Zhou, Z. Shao, *Adv. Funct. Mater.* **2021**, *31*, 2101556.

[30] K. Liu, Y. Li, R. Zhang, M. Wu, B. Huang, T. Zhao, *ACS Appl. Energy Mater.* **2019**, *2*, 6332-6340.

[31] J. Zhang, C. Wang, M. Zheng, M. Ye, H. Zhai, J. Li, G. Tan, X. Tang, X. Sun, *Nano Energy* **2022**, *102*, 107672.

[32] M. He, Z. Cui, C. Chen, Y. Li, X. Guo, *J. Mater. Chem. A* **2018**, *6*, 11463-11470.

[33] B. Zhao, W. Ma, B. Li, X. Hu, S. Lu, X. Liu, Y. Jiang, J. Zhang, *Nano Energy* **2022**, *91*, 106643.

[34] D. Zhou, G.-X. Ren, N. Zhang, P.-F. Yu, H. Zhang, S. Zheng, Z.-W. Tian, S.-Y. Du, J.-X. Chen, X.-S. Liu, *ACS Appl. Energy Mater.* **2021**, *4*, 2873-2880.

[35] K. Liu, R. Zhang, M. Wu, H. Jiang, T. Zhao, *J. Power Sources* **2019**, *433*, 226691.

[36] Y. Zou, H. Zheng, S. Wu, ZehuanHei, H. Liu, H. Duan, *Mater. Lett.* **2021**, *297*, 129959.

[37] S. Guo, T.-T. Wu, Y.-G. Sun, S.-D. Zhang, B. Li, H.-S. Zhang, M.-Y. Qi, X.-H. Liu, A.-M. Cao, L.-J. Wan, *Adv. Funct. Mater.* **2022**, *32*, 2201498.

[38] X. He, F. Yan, M. Gao, Y. Shi, G. Ge, B. Shen, J. Zhai, *ACS Appl. Mater. Interfaces* **2021**, *13*, 42212-42219.

[39] G. V. Alexander, O. V. Sreejith, M. S. Indu, R. Murugan, *ACS Appl. Energy Mater.* **2020**, *3*, 9010-9017.

[40] Z. Wan, K. Shi, Y. Huang, L. Yang, Q. Yun, L. Chen, F. Ren, F. Kang, Y.-B. He, *J. Power Sources* **2021**, *505*, 230062.

[41] C. Wang, Y. Gong, B. Liu, K. Fu, Y. Yao, E. Hitz, Y. Li, J. Dai, S. Xu, W. Luo, E. D. Wachsman, L. Hu, *Nano Lett.* **2017**, *17*, 565-571.

[42] Z. Bi, Q. Sun, M. Jia, M. Zuo, N. Zhao, X. Guo, *Adv. Funct. Mater.* **2022**, *32*, 2208751.

[43] S.-S. Chi, Y. Liu, N. Zhao, X. Guo, C.-W. Nan, L.-Z. Fan, *Energy Storage Mater.* **2019**, *17*, 309-316.

[44] H. Huo, J. Gao, N. Zhao, D. Zhang, N. G. Holmes, X. Li, Y. Sun, J. Fu, R. Li, X. Guo, X. Sun, *Nature Communications* **2021**, *12*, 176.

[45] Z. Bi, W. Huang, S. Mu, W. Sun, N. Zhao, X. Guo, *Nano Energy* **2021**, *90*, 106498.

[46] X. Zhang, Q. Xiang, S. Tang, A. Wang, X. Liu, J. Luo, *Nano Lett.* **2020**, *20*, 2871-2878.

[47] Z. Cheng, M. Xie, Y. Mao, J. Ou, S. Zhang, Z. Zhao, J. Li, F. Fu, J. Wu, Y. Shen, D. Lu, H. Chen, *Adv. Energy Mater.* **2020**, *10*, 1904230.

[48] Z. Lu, J. Yu, J. Wu, M. B. Effat, S. C. T. Kwok, Y. Lyu, M. M. F. Yuen, F. Ciucci, *Energy Storage Mater.* **2019**, *18*, 311-319.

[49] H. Zheng, S. Wu, R. Tian, Z. Xu, H. Zhu, H. Duan, H. Liu, *Advanced Functional Materials* **2020**, *30*, 1906189.

[50] H. Zhang, G. Paggiaro, F. Okur, J. Huwiler, C. Cancellieri, L. P. H. Jeurgens, D. Chernyshov, W. van Beek, M. V. Kovalenko, K. V. Kravchyk, *ACS Appl. Energy Mater.* **2023**, *6*, 6972-6980.

[51] M. J. Wang, R. Choudhury, J. Sakamoto, *Joule* **2019**, *3*, 2165-2178.

[52] J.-F. Wu, B.-W. Pu, D. Wang, S.-Q. Shi, N. Zhao, X. Guo, X. Guo, *ACS Appl. Mater. Interfaces* **2019**, *11*, 898-905.

[53] A. Sharafi, E. Kazyak, A. L. Davis, S. Yu, T. Thompson, D. J. Siegel, N. P. Dasgupta, J. Sakamoto, *Chem. Mater.* **2017**, *29*, 7961-7968.

[54] L. Cheng, E. J. Crumlin, W. Chen, R. Qiao, H. Hou, S. Franz Lux, V. Zorba, R. Russo, R. Kostecki, Z. Liu, K. Persson, W. Yang, J. Cabana, T. Richardson, G. Chen, M. Doeff, *Phys. Chem. Chem. Phys.* **2014**, *16*, 18294-18300.

[55] Y. Li, X. Chen, A. Dolocan, Z. Cui, S. Xin, L. Xue, H. Xu, K. Park, J. B. Goodenough, *J. Am. Chem. Soc.* **2018**, *140*, 6448-6455.

[56] H. Huo, Y. Chen, N. Zhao, X. Lin, J. Luo, X. Yang, Y. Liu, X. Guo, X. Sun, *Nano Energy* **2019**, *61*, 119-125.

[57] M. Motoyama, Y. Tanaka, T. Yamamoto, N. Tsuchimine, S. Kobayashi, Y. Iriyama, *ACS Appl. Energy Mater.* **2019**, *2*, 6720-6731.

[58] S. Shin, J. Lee, T. H. Shin, S. Lee, *Journal of Energy Chemistry* **2024**, *92*, 394-403.

[59] Y. Ruan, Y. Lu, X. Huang, J. Su, C. Sun, J. Jin, Z. Wen, *J. Mater. Chem. A* **2019**, *7*, 14565-14574.

[60] M. Cai, J. Jin, T. Xiu, Z. Song, M. E. Badding, Z. Wen, *Energy Storage Mater.* **2022**, *47*, 61-69.

[61] M. Cai, Y. Lu, J. Su, Y. Ruan, C. Chen, B. V. R. Chowdari, Z. Wen, *ACS Appl. Mater. Interfaces* **2019**, *11*, 35030-35038.

[62] H. Duan, W.-P. Chen, M. Fan, W.-P. Wang, L. Yu, S.-J. Tan, X. Chen, Q. Zhang, S. Xin, L.-J. Wan, Y.-G. Guo, *Angew. Chem. Int. Ed.* **2020**, *59*, 12069-12075.

[63] J. Gao, W. Guo, Y. Yin, Z. Sun, B. Zhao, F. Shen, X. Han, *Mater. Lett.* **2020**, *280*, 128543.

[64] M. Jia, Z. Bi, C. Shi, N. Zhao, X. Guo, *J. Power Sources* **2021**, *486*, 229363.
[65] M. M. Besli, C. Usubelli, M. Metzger, V. Pande, K. Harry, D. Nordlund, S. Sainio, J. Christensen, M. M. Doeff, S. Kuppan, *ACS Appl. Mater. Interfaces* **2020**, *12*, 20605-20612.
[66] W. Ji, B. Luo, Q. Wang, G. Yu, Z. Liu, Z. Zhao, R. Zhao, S. Wang, X. Wang, B. Zhang, J. Zhang, F. Hou, J. Liang, *Adv. Energy Mater.* **2023**, *13*, 2300165.
[67] Z. Qin, Y. Xie, X. Meng, D. Qian, C. Shan, D. Mao, G. He, Z. Zheng, L. Wan, Y. Huang, *Chem. Eng. J.* **2022**, *447*, 137538.
[68] X. Ma, Y. Xu, *Electrochim. Acta* **2023**, *441*, 141789.
[69] L. Chen, Y. Su, J. Zhang, H. Zhang, B. Fan, G. Shao, M. Zhong, C.-A. Wang, *ACS Appl. Mater. Interfaces* **2021**, *13*, 37082-37090.
[70] F. Okur, H. Zhang, J. F. Baumgärtner, J. Sivavec, M. Klimpel, G. P. Wasser, R. Dubey, L. P. H. Jeurgens, D. Chernyshov, W. van Beek, K. V. Kravchyk, M. V. Kovalenko, *Adv. Sci.* **2025**, *12*, 2412370.
[71] Z. Jiang, S. Wang, X. Chen, W. Yang, X. Yao, X. Hu, Q. Han, H. Wang, *Adv. Mater.* **2020**, *32*, 1906221.
[72] E. Yi, H. Shen, S. Heywood, J. Alvarado, D. Y. Parkinson, G. Chen, S. W. Sofie, M. M. Doeff, *ACS Appl. Energy Mater.* **2020**, *3*, 170-175.
[73] G. T. Hitz, D. W. McOwen, L. Zhang, Z. Ma, Z. Fu, Y. Wen, Y. Gong, J. Dai, T. R. Hamann, L. Hu, E. D. Wachsman, *Mater. Today* **2019**, *22*, 50-57.
[74] C. Yang, L. Zhang, B. Liu, S. Xu, T. Hamann, D. McOwen, J. Dai, W. Luo, Y. Gong, E. D. Wachsman, L. Hu, *Proceedings of the National Academy of Sciences* **2018**, *115*, 3770-3775.
[75] S. Xu, D. W. McOwen, C. Wang, L. Zhang, W. Luo, C. Chen, Y. Li, Y. Gong, J. Dai, Y. Kuang, C. Yang, T. R. Hamann, E. D. Wachsman, L. Hu, *Nano Lett.* **2018**, *18*, 3926-3933.
[76] H. Zhang, F. Okur, C. Cancellieri, L. P. H. Jeurgens, A. Parrilli, D. T. Karabay, M. Nesvadba, S. Hwang, A. Neels, M. V. Kovalenko, K. V. Kravchyk, *Adv. Sci.* **2023**, *10*, 2205821.
[77] H. Zhang, R. Dubey, M. Inniger, F. Okur, R. Wullich, A. Parrilli, D. T. Karabay, A. Neels, K. V. Kravchyk, M. V. Kovalenko, *Cell Rep. Phys. Sci.* **2023**, *4*, 101473.
[78] H. Zhang, F. Okur, B. Pant, M. Klimpel, S. Butenko, D. T. Karabay, A. Parrilli, A. Neels, Y. Cao, K. V. Kravchyk, M. V. Kovalenko, *ACS Appl. Mater. Interfaces* **2024**, *16*, 12353-12362.
[79] H. Xie, C. Yang, Y. Ren, S. Xu, T. R. Hamann, D. W. McOwen, E. D. Wachsman, L. Hu, *Nano Lett.* **2021**, *21*, 6163-6170.
[80] G. V. Alexander, C. Shi, J. O'Neill, E. D. Wachsman, *Nat. Mater.* **2023**, *22*, 1136-1143.
[81] K. Fu, Y. Gong, G. T. Hitz, D. W. McOwen, Y. Li, S. Xu, Y. Wen, L. Zhang, C. Wang, G. Pastel, J. Dai, B. Liu, H. Xie, Y. Yao, E. D. Wachsman, L. Hu, *Energy & Environmental Science* **2017**, *10*, 1568-1575.
[82] B. Liu, L. Zhang, S. Xu, D. W. McOwen, Y. Gong, C. Yang, G. R. Pastel, H. Xie, K. Fu, J. Dai, C. Chen, E. D. Wachsman, L. Hu, *Energy Storage Mater.* **2018**, *14*, 376-382.
[83] https://www.dnkpower.com/wp-content/uploads/2022/08/SANYO-NCR18650GA-Datasheet.pdf, **2024**.

[84] J. B. Quinn, T. Waldmann, K. Richter, M. Kasper, M. Wohlfahrt-Mehrens, *J. Electrochem. Soc.* **2018**, *165*, A3284.
